# The E301R protein of African swine fever virus functions as a sliding clamp involved in viral genome replication

**DOI:** 10.1128/mbio.01645-23

**Published:** 2023-09-29

**Authors:** Su Li, Hailiang Ge, Yanhua Li, Kehui Zhang, Shaoxiong Yu, Hongwei Cao, Yanjin Wang, Hao Deng, Jiaqi Li, Jingwen Dai, Lian-Feng Li, Yuzi Luo, Yuan Sun, Zhi Geng, Yuhui Dong, Heng Zhang, Hua-Ji Qiu

**Affiliations:** 1 State Key Laboratory for Animal Disease Control and Prevention, National African Swine Fever Para-Reference Laboratory, National High-Containment Facilities for Animal Disease Control and Prevention, Harbin Veterinary Research Institute, Chinese Academy of Agricultural Sciences, Harbin, China; 2 College of Animal Sciences, Yangtze University, Jingzhou, China; 3 Multidiscipline Research Center, Institute of High Energy Physics, Chinese Academy of Sciences, Beijing, China; University of Calgary, Calgary, Canada

**Keywords:** African swine fever virus, E301R protein, sliding clamp, DNA polymerase, genomic replication

## Abstract

African swine fever virus (ASFV) is a complex nucleocytoplasmic, large DNA virus that infects both domestic pigs and wild boar, but little is known about the process of genomic replication. The E301R protein (pE301R) from ASFV was previously predicted as a proliferating cell nuclear antigen (PCNA)-like protein through clamping DNA polymerase to the DNA duplex, but its exact structure and functions remain uncharacterized. Here, the crystal structure of pE301R revealed that it is composed of structurally similar head and tail domains and shares significant structural similarities to the DNA polymerase processivity factors, including sliding clamp and eukaryotic PCNA. More specifically, we demonstrated that pE301R exhibited multiple oligomeric states (with dimers and tetramers dominant), and the tetramers are consistent with the ring-shaped homotetramers in a head-to-tail manner generated by crystal packing. We also showed that pE301R interacted with the ASFV genome and viral DNA polymerase O174L. Furthermore, knockdown of *E301R* by specific small interfering RNAs (siRNAs) significantly decreased the virus genomic replication. Interestingly, the downregulation of PCNA by siRNAs significantly decreased the cell viability, whereas the inhibitory effect was reversed by pE301R overexpression. Notably, we demonstrated that overexpression of PCNA partially restored ASFV replication upon transfection of siRNAs targeting *E301R*. More importantly, T2 amino alcohol, a PCNA-specific inhibitor, markedly inhibited ASFV replication at the stage of viral genome replication. Taken together, we revealed that pE301R functions as a sliding clamp in ASFV genomic replication and can be used as a potential antiviral target.

**IMPORTANCE:**

Sliding clamp is a highly conserved protein in the evolution of prokaryotic and eukaryotic cells. The sliding clamp is required for genomic replication as a critical co-factor of DNA polymerases. However, the sliding clamp analogs in viruses remain largely unknown. We found that the ASFV E301R protein (pE301R) exhibited a sliding clamp-like structure and similar functions during ASFV replication. Interestingly, pE301R is assembled into a unique ring-shaped homotetramer distinct from sliding clamps or proliferating cell nuclear antigens (PCNAs) from other species. Notably, the *E301R* gene is required for viral life cycle, but the pE301R function can be partially restored by the porcine PCNA. This study not only highlights the functional role of the ASFV pE301R as a viral sliding clamp analog, but also facilitates the dissection of the complex replication mechanism of ASFV, which provides novel clues for developing antivirals against ASF.

## INTRODUCTION

African swine fever (ASF) is a severe porcine viral disease with an acute hemorrhagic fever and a high-case fatality, threatening the global pig industry. The disease is an epidemic in Africa, Eastern Europe, and Asia (1). Currently, no vaccines are available for ASF except in Vietnam. African swine fever virus (ASFV), the causative agent of ASF, is a large enveloped double-stranded DNA virus and the sole member of the family *Asfarviridae* (2). The ASFV genome is approximately 170 to 194 kilobase pairs and contains more than 150 open reading frames (ORFs), encoding viral proteins involved in genome replication, transcription and translation, virion assembly, and immune evasion.

In general, DNA polymerase processivity factors (PFs), also known as sliding clamps, are associated with their cognate DNA polymerases on the template during replication and functionally and structurally similar among various organisms, including bacteria, *Archaea*, bacteriophages, and eukaryotes. Sliding clamps usually display the homodimers or homotrimers that form a ring-shaped sliding clamp around the DNA (3 – 5). The siding clamp functions by binding and tethering the polymerase to the DNA to increase the polymerase processibility during the elongation of the leading and lagging strands. In vaccinia virus, a distant relative of ASFV, the G8R protein was predicted with a proliferating cell nuclear antigen (PCNA)-like structure by homology modeling, and virus replication can be inhibited by the PCNA-specific inhibitors, but the PCNA-like structure and function of the viral protein have not been clarified by *in vitro* biochemical and functional tests (6). The herpesviruses belong to large DNA viruses, and their polymerase PFs exhibit diverse molecular assemblies. The Epstein-Barr virus (EBV) PF BMRF1 can form a ring-shaped tetramer structure (7), while the herpes simplex virus type 1 (HSV-1) UL42 protein binds directly to DNA as a monomer (8). The human cytomegalovirus (HCMV) UL44 forms a dimers and possesses DNA binding activities (9). A recent study shows that the monkeypox virus A22 and E4 proteins can form a circular presliding clamp that binds the DNA polymerase and viral genome to maintain sustainable DNA synthesis (10). Thus, DNA viruses utilize different strategies to maintain the continuous functionality of DNA polymerase. However, the sliding clamp of DNA polymerase encoded by ASFV remains to be defined.

Several proteins encoded by the ASFV genome have been predicted to be involved in viral genome replication (11). The *C962R* gene encodes an NTPase that shows certain homology with the vaccinia virus D5 protein, which is required for DNA replication and might function as the replication fork (12, 13). The DNA polymerase X (pO174L) and the DNA polymerase type B (pG1211R) of ASFV will likely participate in viral genome replication. In addition, the F1055L protein shares a high sequence similarity to the herpesviral UL9 protein, which binds to the origin of replication and fusion with a putative DNA primase (14). Moreover, other ASFV-encoded proteins were predicted to participate in DNA replication or repair, including DNA topoisomerase type II (pP1192R) (15, 16) and DNA ligase (pNP419L) (17).

The E301R protein (pE301R) is a non-structural protein of ASFV encoded by the *E301R* gene. We speculated that pE301R is involved in the virus DNA replication as a PCNA-like protein that may function in clamping DNA polymerase to the DNA duplex (18, 19). However, its intact role in DNA replication of the ASFV life cycle has not been experimentally validated up to now. This study comprehensively characterized the protein involvement in ASFV replication and infection by structure-function analysis. We demonstrate that the protein displays a ring-shaped homotetramer and functions as a viral analog of the sliding clamp.

## RESULTS

### pE301R is highly conserved among different ASFV isolates

**Fig 1:**
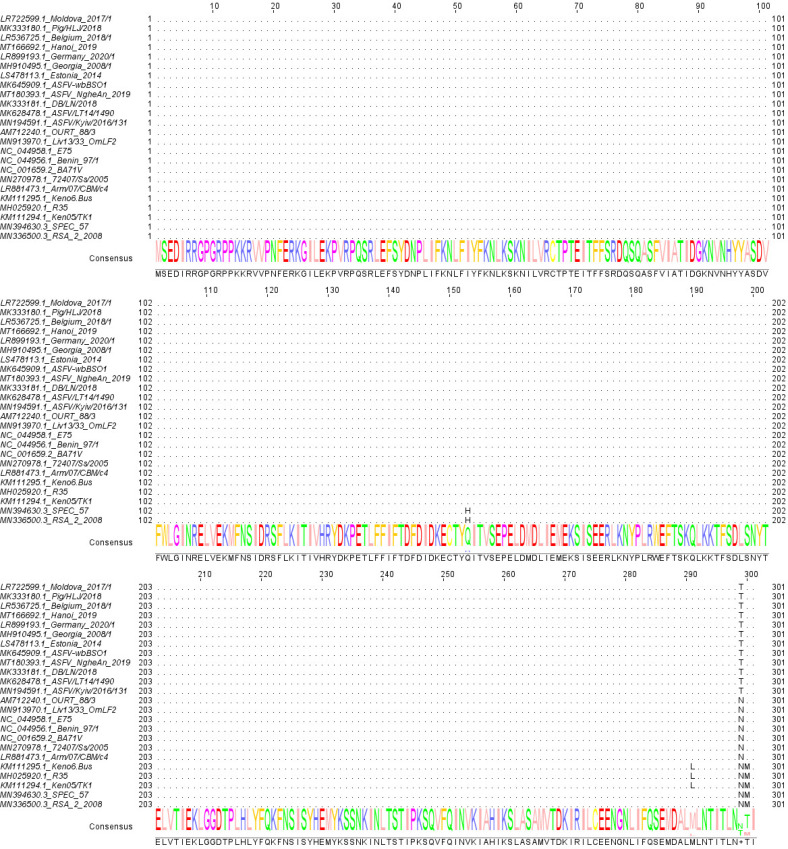
The E301R protein (pE301R) is a highly conserved viral protein among different African swine fever virus (ASFV) isolates. Twenty-four pE301R sequences of ASFV were collected from the GenBank database to conduct a multiple sequence alignment using the Clustal W algorithm. Dots represent identical amino acids, and capital letters represent the different ones.

The *E301R* gene is located at the 3′ end of the left strand of the ASFV genome between the *E423R* and *E146L* genes. The *E301R* gene of the ASFV Pig/HLJ/2018 (ASFV-WT) strain is located between nucleotide positions 164265 and 165170 in the genome. The amino acid (aa) sequences of pE301R from 24 ASFV isolates were analyzed by multiple sequence alignment by Clustal W (https://www.genome.jp/tools-bin/clustalw) and embellished using the Jalview software (version 2.11.1.4). We found that the aa sequence identities vary from 99% to 100% (Fig. 1), indicating the high-level conservation of pE301R among various ASFV isolates.

### *E301R* is transcribed at the middle stage of the ASFV life cycle and mainly localized in the viral factories of the cytoplasm

**Fig 2:**
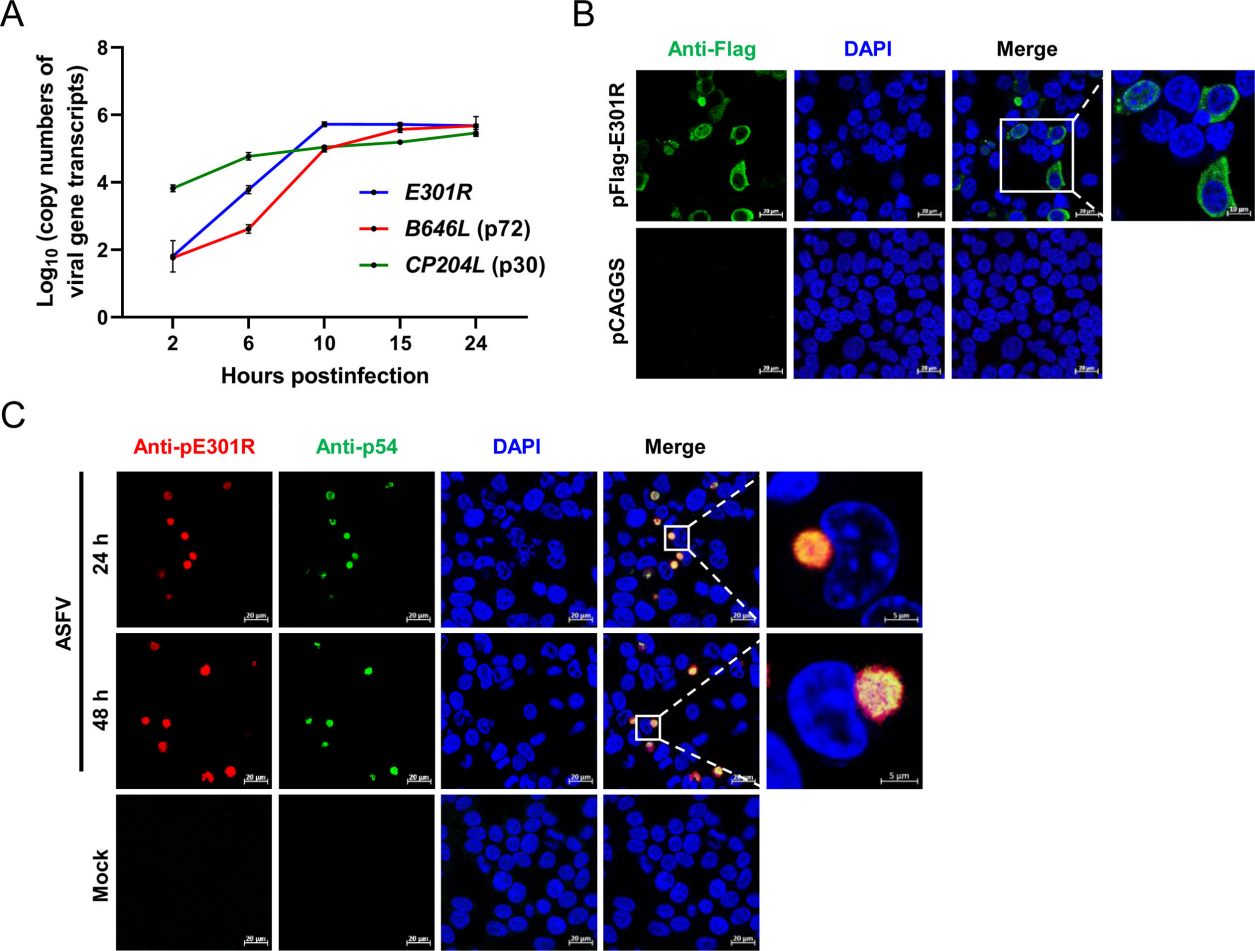
Expression characteristics of the E301R protein (pE301R) of African swine fever virus (ASFV). (**A**) The *E301R* gene transcriptional dynamics. Primary porcine alveolar macrophages (PAMs) were infected with the African swine fever virus (ASFV) Pig/HLJ/2018 strain (ASFV-WT) at a multiplicity of infection (MOI) of 1, and the viral gene transcription was quantified at 2, 6, 10, 15, and 24 hours postinfection by reverse transcription-quantitative PCR using the primers targeting *E301R*, *CP204L*, and *B646L*. (**B**) Intracellular localization of ectopic expression of pE301R. HEK293T cells were transfected with pFlag-E301R (1 µg) for 24 hours. The cells were fixed and probed with a mouse anti-Flag monoclonal antibody and the nuclear marker 4,6-diamidino-2-phenylindole (DAPI), and then examined by laser confocal microscopy. Scale bars, 20 µm. (**C**) Subcellular localization of pE301R in the ASFV-infected cells. PAMs were infected with the ASFV-WT at an MOI of 1 for 24 and 48 hours. The cells were fixed and incubated with home-made mouse anti-pE301R or swine anti-p54 polyclonal antibodies. The subcellular localization of pE301R or p54 was visualized by laser confocal microscopy. Scale bars, 20 µm.

To evaluate the transcription kinetics of *E301R*, primary porcine alveolar macrophages (PAMs) were infected with ASFV-WT at a multiplicity of infection (MOI) of 1 and subjected to total RNA extraction and reverse transcription-quantitative PCR (RT-qPCR) at 2, 6, 10, 15, and 24 hours postinfection (hpi) (approximately one cycle of replication for ASFV) (20). The mRNA transcription of *E301R* was lower than the early-transcribed gene *CP204L* within 2 hpi, then markedly increased, and reached a peak at 10 hpi (Fig. 2A). The transcription kinetics of *E301R* is earlier than the late-transcribed gene *B646L*, indicating that the *E301R* gene is transcribed at the middle stage of the ASFV life cycle.

To determine the subcellular localization of pE301R in cells, HEK293T cells were transfected with pFlag-E301R expressing the Flag-tagged pE301R in the pCAGGS vector and analyzed by laser confocal microscopy. The results showed that the protein was mainly localized in the cytoplasm, occasionally, in the nucleus (Fig. 2B). To investigate the subcellular localization of pE301R in the ASFV-infected cells, PAMs were infected with ASFV at an MOI of 1 for 24 and 48 hours. The subcellular localization of pE301R was visualized by an immunofluorescence assay using homemade mouse anti-E301R polyclonal antibodies (PAbs). The fluorescence of pE301R was observed mainly within the perinuclear viral factories, which were co-localized with the ASFV p54 protein (a marker of viral factories [21]) (Fig. 2C).

### pE301R displays a sliding clamp-like structure

**Fig 3:**
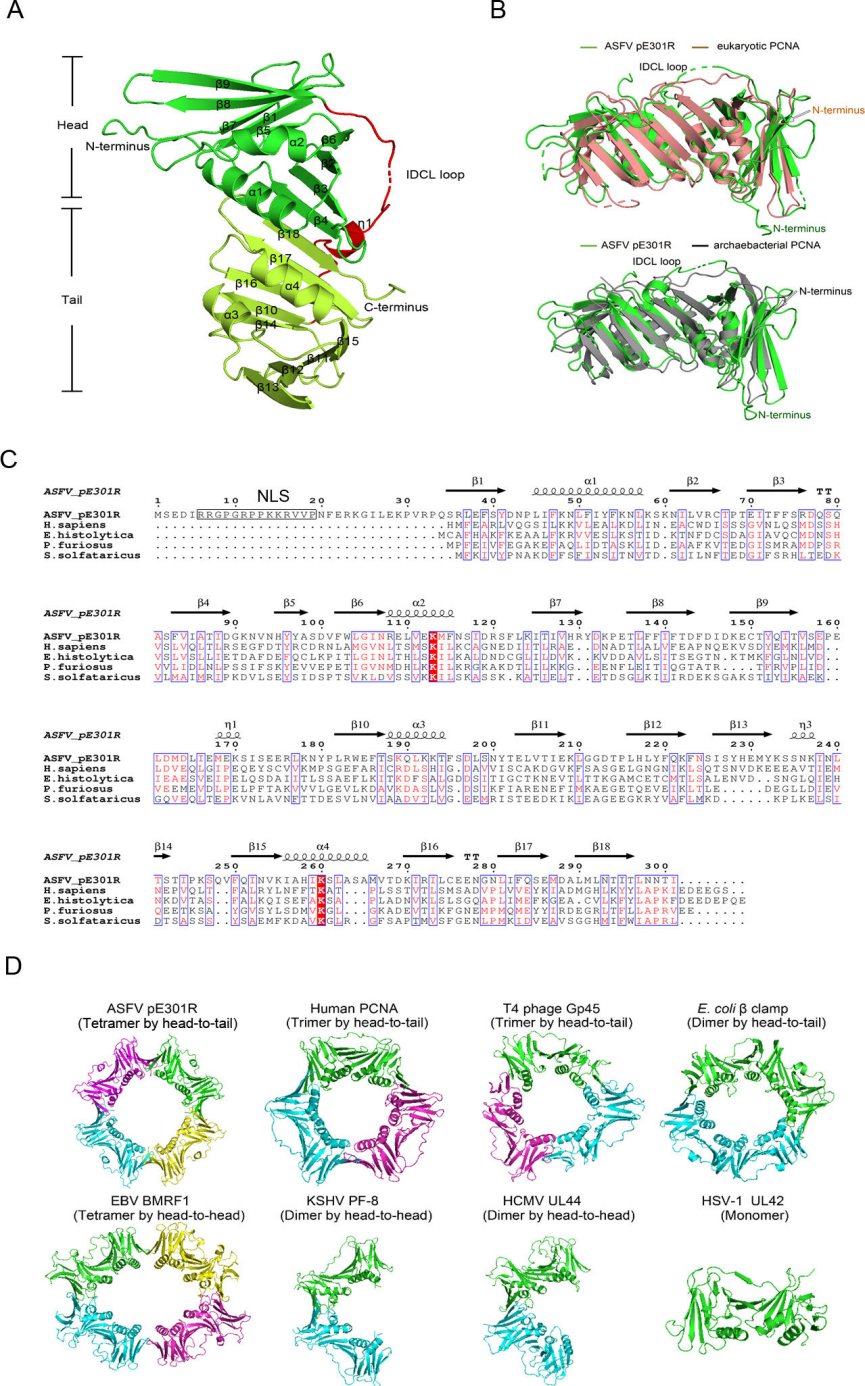
Overview of the E301R protein (pE301R) structure. (**A**) Overall crystal structure of pE301R at 2.10-Å resolution. The two globular domains are colored green and lemon, respectively, connected by the IDCL loop and highlighted in red. (**B**) Structural superimposition of ASFV pE301R (green) with the PCNAs from *Homo sapiens* (PDB ID: 1VYM, brown) and *Pyrococcus furiosus* (PDB ID: 3A2F, gray). (**C**) Structure-based sequence alignment of pE301R with its representative homologs. The sequence alignment of the DNA sliding clamps from *H. sapiens*, *Entamoeba histolytica*, *P. furiosus*, and *Sulfolobus solfataricus* was performed using Clustal X (version 1.81) and ESPript 3. The conserved residues are boxed in blue. Identical and low-conserved residues are highlighted in red background and red letters, respectively. The predicted NLS (N-terminus) and NES (C-terminus) are highlighted in black boxes. (**D**) Oligomerization architecture comparison of the ASFV pE301R with representative analogs, including the DNA sliding clamps from herpesviruses, bacteriophages, *Escherichia coli*, and *H. sapiens*.

To gain insights into pE301R functions, we determined its crystal structure using the single-wavelength anomalous dispersion method with the selenomethionine (Se-Met)-labeled proteins (Table 1). The residues 15–162 and 165–301 could be observed in the density map that was built in the final model. The structure contains four α-helices (α1–4) and 18 β-sheets (β1–18), which could be divided into two globular similar subdomains (named as the head domain: *α*1–2 and β1–9 and the tail domain: α3–4/β10–18). The head and tail domains are connected by an inter-domain connecting loop (IDCL) mixed with a short helix (η1) (Fig. 3A).

**TABLE 1:** X-ray data collection and refinement statistics

Data collection	Se-Met pE301R	Native pE301R
Beamline	SSRF^*a*^ 18U1	SSRF^*a*^ 17U1
Wavelength (Å)	0.9788	0.9788
Space group	*I*4	*I*4
Unit-cell parameters	*a* = 107.0 Å, *b* = 107.0 Å, *c* = 75.4 Å, α = β = γ = 90°	*a* = 107.6 Å, *b* = 107.6 Å, *c* = 75.3 Å, α = β = γ = 90°
Resolution (Å)	2.70 (2.75–2.70)^*b*^	2.10 (2.15–2.10)^*b*^
Number of unique reflections	11 911 (595)	25 053 (1855)
Completeness (%)	100.0 (100.0)	99.5 (100.0)
Redundancy	12.5 (11.7)	13.4 (14.1)
Mean *I*/ơ (*I*)	28.29 (2.00)	32.30 (3.43)
Molecules in asymmetric unit	1	1
*R*_merge_ (%)	7.5 (87.1)	4.4 (83.7)
*R*_meas_ (%)	7.8 (91.0)	4.6 (91.0)
CC_1/2_	99.8 (87.5)	100 (87.4)
Structure refinement		
Reflections used in refinement		25,050
Resolution range (Å)		19.32–2.10
*R*_work_/*R*_free_ (%)		22.6/26.7
Protein atoms		2270
Protein residues		274
Waters		67
Average B factor (Å^2^)		
Ramachandran plot (%)		
Most favored		94.0
Allowed		5.6
Disallowed		0.4
r.m.s.d.		
Bond lengths (Å)		0.010
Bond angles (°)		1.143

^*a*^ Shanghai Synchrotron Radiation Facility.

^*b*^ The values in parenthesis means those for the highest resolution shell.

A DALI homolog search (http://ekhidna.biocenter.helsinki.fi/dali_server) shows that pE301R has significant structural similarity with eukaryotic and archaebacterial PCNAs, although there are only 10%–15% aa sequence identities among them. The structural analogs include the eukaryotic PCNAs from *Homo sapiens*, *Entamoeba histolytica*, and *Arabidopsis thaliana* (PDBs: 6S1O, 3P91, and 6O09, with *Z*-scores of 23.2–23.7 and with root mean square deviation [r.m.s.d.] of 2.5–2.9 Å), and archaebacterial PCNAs including *Pyrococcus furiosus* and *Sulfolobus solfataricus* (PDB IDs: 3A2F and 2HII, with *Z*-scores of 20.2–22.5 and with r.m.s.d. of 2.6–3.0 Å). Structural comparisons of pE301R with the human and *P. furiosus* PCNAs revealed the major differences are their various conformations of the IDCL loops, as well as the long N-terminal loop (residues 1–36) that is absent in eukaryotic and archaebacterial PCNAs (Fig. 3B and C). Moreover, pE301R also has notable similarities with several viral DNA polymerase PFs from herpesviruses, including HSV-1 UL42 (PDB ID: 1DML), HCMV UL44 (PDB ID: 1T6L), Kaposi's sarcoma-associated herpesvirus (KSHV) PF-8 (PDB ID: 3HSL), and EBV BMRF1 (PDB ID: 2Z0L) (with *Z*-scores of 12.7–15.3 and with r.m.s.d. of 3.1–3.7 Å) (Fig. 3D).

### pE301R may assemble into a ring-shaped homotetramer

**Fig 4:**
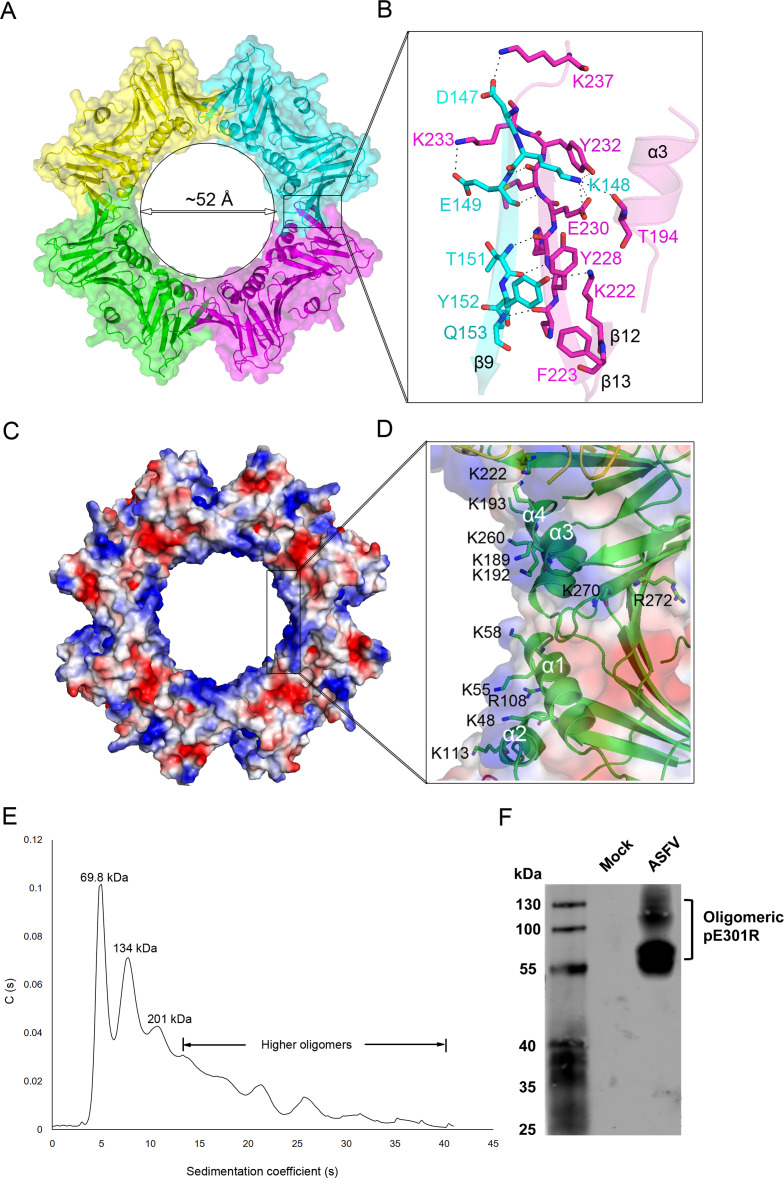
Structural characterization of the ring-shaped homotetramer of E301R protein (pE301R). (**A**) A ring-shaped homotetramer can be generated with symmetry-related molecules in a head-to-tail manner. The four protomers are shown in magenta, green, yellow, and cyan, respectively. (**B**) Contact analysis of the two protomers in the interface. The homotetramer is mediated by the interactions mainly between β9 in one subunit and β13 in another one via multiple hydrogen bonds as well as hydrophobic contacts. The assembly of the tetramer produces a central channel of approximately 52 Å in diameter. (**C**) The molecular surface representation of the ring-shaped homotetramer (blue, +7.3 KT; red, −7.3 KT), colored by its local electrostatic potential. These monomers are arranged in such a way that the inner cavity of the tetrameric ring has a positively charged surface that provides a favorable electrostatic environment for nonspecific interactions with the sugar-phosphate backbone of DNA. (**D**) Distribution of the basic residues (shown as sticks) in the inner face of the ring (only one protomer is shown). (**E**) The oligomeric state of recombinant pE301R in solution was dissected by analytical ultracentrifugation. (**F**) Detection of pE301R expression in the ASFV-infected primary porcine alveolar macrophages by western blotting analysis. PAMs were infected with ASFV-WT at a multiplicity of infection of 1 for 48 hours. The cells were lysed with NP-40 and examined by native-PAGE and western blotting analysis using anti-pE301R polyclonal antibodies.

Inspection of the crystal packing of the pE301R structure revealed that it could form a homotetramer ring with the neighboring molecules in a head-to-tail manner, around a crystallographic fourfold axis with pseudo-eightfold symmetry. The assembly of the tetramer produces a central channel of approximately 52 Å in diameter (Fig. 4A). The homotetramer is mediated by the interactions mainly between β9 in one subunit and β13 in another one via multiple hydrogen bonds/salt bridges as well as hydrophobic contacts (Fig. 4B). For example, the side chain of K148 of one subunit forms two salt bridges with the side chains of E230 of neighboring subunit, while Y152 of one subunit generates hydrophobic aromatic stackings with Y228 and F223 of neighboring subunit, to stabilize the contacts between the two protomers. The inner surface of the ring is composed of the 16 helices (α1–4 from each of the four protomers) (Fig. 4C and D) that are oriented to form a central hole that can encircle double-stranded DNA and allow its sliding like PCNA. Inspection of the charge distribution of the tetrameric ring revealed that its inner cavity has a positively charged surface (Fig. 4D) distributed with many basic residues (lysine and arginine). Such an arrangement may provide a favorable electrostatic environment for potential nonspecific interactions with the DNA backbone.

To verify whether the above tetramers form exists in solution, the analytical ultracentrifugation (AUC) analysis of recombinant pE301R showed that pE301R exhibited multiple oligomeric forms in solution. The dimers (around 69.8 kDa) and tetramers (around 134 kDa) are the predominant oligomers, with a small portion of hexamers (around 201 kDa) and higher oligomers (Fig. 4E). To evaluate the oligomeric structure of pE301R in the context of ASFV infection, PAMs were infected with ASFV-WT at an MOI of 1 for 48 hours, and the cell lysates were subjected to native-PAGE and western blotting analysis using the anti-pE301R PAbs. As shown in Fig. 4F, two major protein bands were observed, with a molecular weight (MW) of around 130 (close to tetramers) and 70 (dimers) kDa, respectively, indicating that pE301R functions as an oligomeric protein in the ASFV-infected cells. Altogether, these results suggest that pE301R can be assembled into a ring-shaped tetramer structure similar to the sliding clamps, or as a dimer structure like the DNA polymerase PFs of several herpesviruses such as HCMV (9).

### pE301R interacts with the ASFV genome and with the viral DNA polymerase O174L

**Fig 5:**
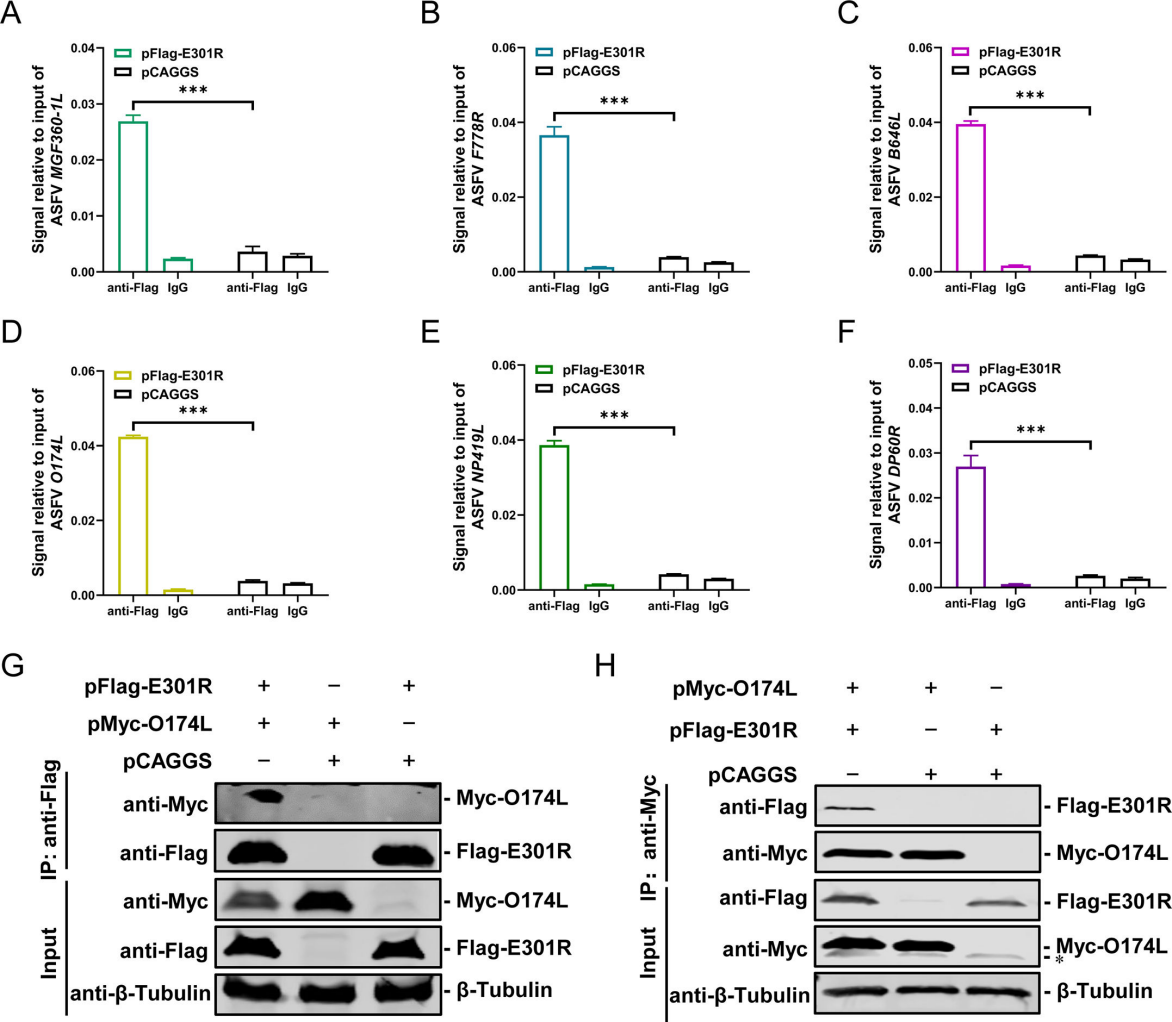
E301R protein (pE301R) interacts with the African swine fever virus (ASFV) genome and the DNA polymerase O174L. (A–F) Chromatin immunoprecipitation-quantitative PCR (ChIP-qPCR) analysis of pE301R and virus genome. HEK293T cells transfected with pFlag-E301R or pCAGGS (negative control) were infected with ASFV-P121 at a multiplicity of infection of 1 for 48 hours. Then the cells were subjected to ChIP-qPCR analysis using anti-Flag polyclonal antibodies (PAbs). Quantitative PCR was used to quantify the different genes of ASFV including *MGF-360-IL*, *F778R*, *B646L*, O174L, *NP419L*, and *DP60R*. (**G and H**) Identification of the interaction between pE301R and pO174L by co-immunoprecipitation assay. HEK293T cells were cotransfected with pFlag-E301R and pMyc-O174L for 48 hours. The cells were lysed, precleared with protein G-agarose, and incubated with anti-Flag M2 (**G**) or anti-c-Myc magnetic beads (**H**). After washing with cold phosphate-buffered saline, the bound proteins were analyzed by western blotting using rabbit anti-Flag or anti-Myc PAbs (1:500). *Non-specific band. ***P <0.001.

To determine whether pE301R functions as a sliding clamp to interact with the ASFV genome, HEK293T cells were transfected with pFlag-E301R or pCAGGS (negative control), followed by infection with ASFV-P121 at an MOI of 1 for 48 hours. Then the cells were subjected to chromatin immunoprecipitation (ChIP) analysis using an anti-Flag monoclonal antibody (MAb). The different genes of the ASFV genome, including *MGF360-1L*, *F778R*, *B646L*, *O174L*, *NP419L*, and *DP60R*, were determined by quantitative PCR (qPCR). Compared with the negative control, the binding of ASFV genes to pE301R demonstrated a substantial increase (Fig. 5A to F), indicating a significant interaction between pE301R and the ASFV genome.

The DNA polymerase maintains its association with the template strand by tethering to the sliding clamps, which moves along with the sliding clamps during DNA synthesis (22, 23). The ASFV *O174L* gene encodes a 174-aa DNA polymerase that belongs to the polymerase X (pol X) family (24). The ASFV pol X is important in maintaining the viral genome and generating ASFV variants and genotypes (25). To investigate whether pE301R displays a sliding clamp-like function by interacting with pO174L, the *E301R*- and *O174L*-expressing plasmids were cotransfected into HEK293T cells for coimmunoprecipitation (Co-IP) assay. The Flag-tagged pE301R interacted with the Myc-tagged pO174L (Fig. 5G), and the Flag-tagged pO174L was coprecipitated with the Myc-tagged pE301R (Fig. 5H), indicating that pE301R displays the characteristic of the sliding clamp.

### The ectopically expressed pE301R restores the cell viability of the PCNA-knockdown cells

**Fig 6:**
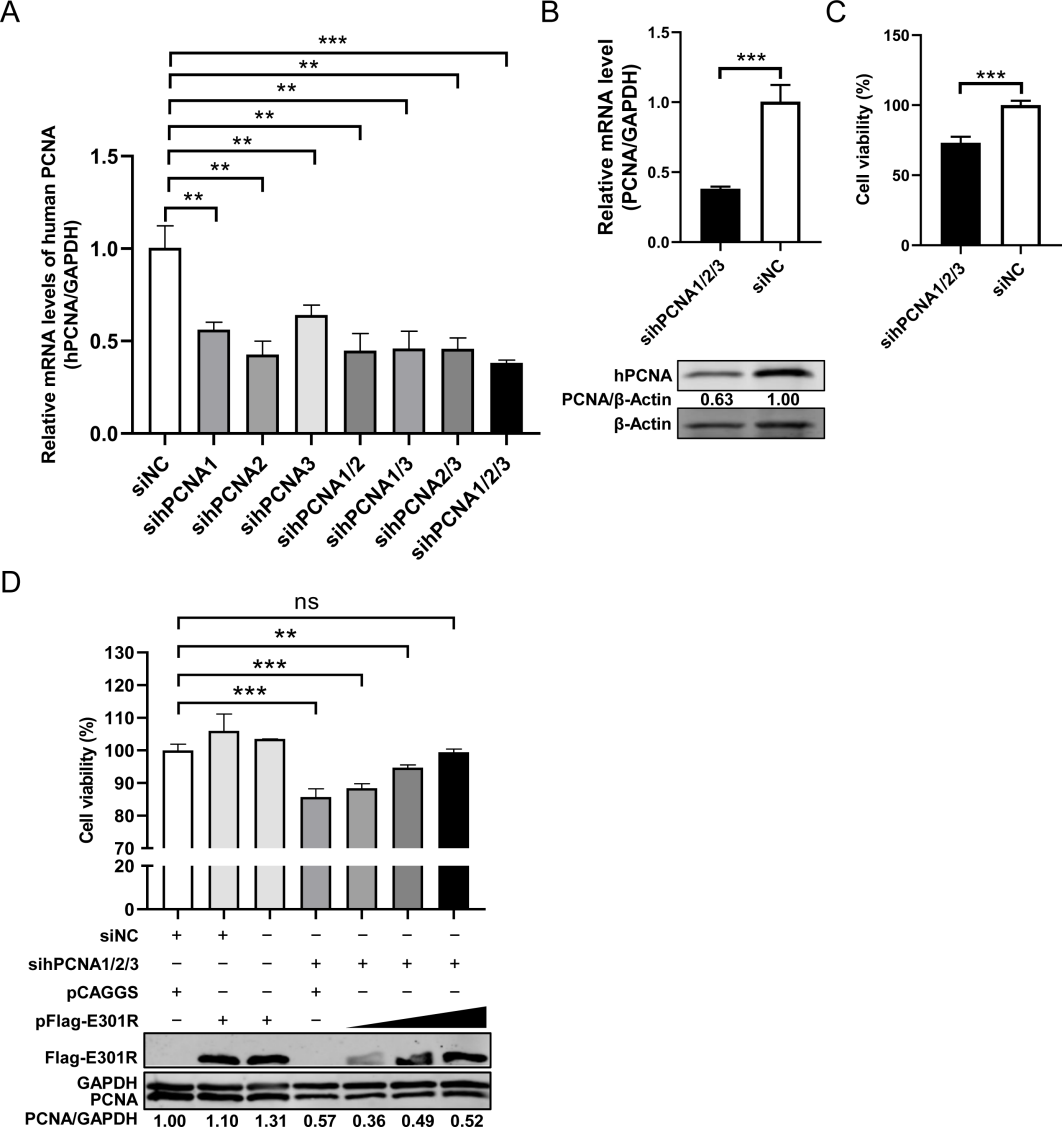
Ectopically expressed E301R protein (pE301R) restores the cell viability of the PCNA-knockdown cells. (**A**) Identification of siRNAs against PCNA with the optimal knockdown efficiency. HEK293T cells transfected with 200 nM single or pooled siRNA(s) against PCNA for 24 hours. The knockdown efficiency of siRNA(s) against PCNA was determined by a relative reverse transcription-quantitative PCR (2^−ΔΔCT^ method). (**B and C**) The effects of *PCNA* knockdown on the cell viability. HEK293T cells transfected with 200 nM pooled siRNAs against the human *PCNA* (sihPCNA1/2/3) or scramble control siRNA (siNC) were harvested at 36 hours posttransfection. Endogenous PCNA was examined by relative reverse transcription-quantitative PCR and immunoblotting with antibodies against the indicated proteins (**B**). The cell viability was determined using the CellTiter-Glo kit (**C**). (**D**) The effects of the ectopically expressed pE301R on the cell viability upon *PCNA* knockdown. HEK293T cells were co-transfected with sihPCNA1/2/3 (200 nM) and pFlag-E301R of various amounts (0, 0.5, 1.0, and 2.0 µg) for 48 hours. The cell viability was determined as described above. The protein expression of PCNA or pE301R was analyzed by western blotting using a mouse anti-PCNA monoclonal antibody or anti-pE301R polyclonal antibodies.**P < 0.01, ***P < 0.001; ns, not significant, *P* > 0.05.

It has been demonstrated that the cellular PCNA is widely involved in molecular events, including chromosomal DNA replication, DNA repair, cell cycle, and apoptosis (26). To investigate whether pE301R exhibits a sliding clamp-like role in cells, HEK293T cells were transfected with the single small interfering RNA (siRNA) or pooled siRNAs (sihPCNAs) (equal amounts of each siRNA) targeting the human PCNA to determine the knockdown efficacy and further examine the cell viability. The data showed that the pooled sihPCNA1/2/3 exhibited the most efficient inhibition of the expression of PCNA (Fig. 6A). Furthermore, knockdown of *PCNA* by sihPCNA1/2/3 resulted in decreased cell viability (Fig. 6B and C), which was restored by the ectopically expressed pE301R in a dose-dependent manner (Fig. 6D), indicating that pE301R exerts a sliding clamp-like function.

### The ectopically expressed PCNA partially restores the ASFV infection with *E301R* knockdown

**Fig 7:**
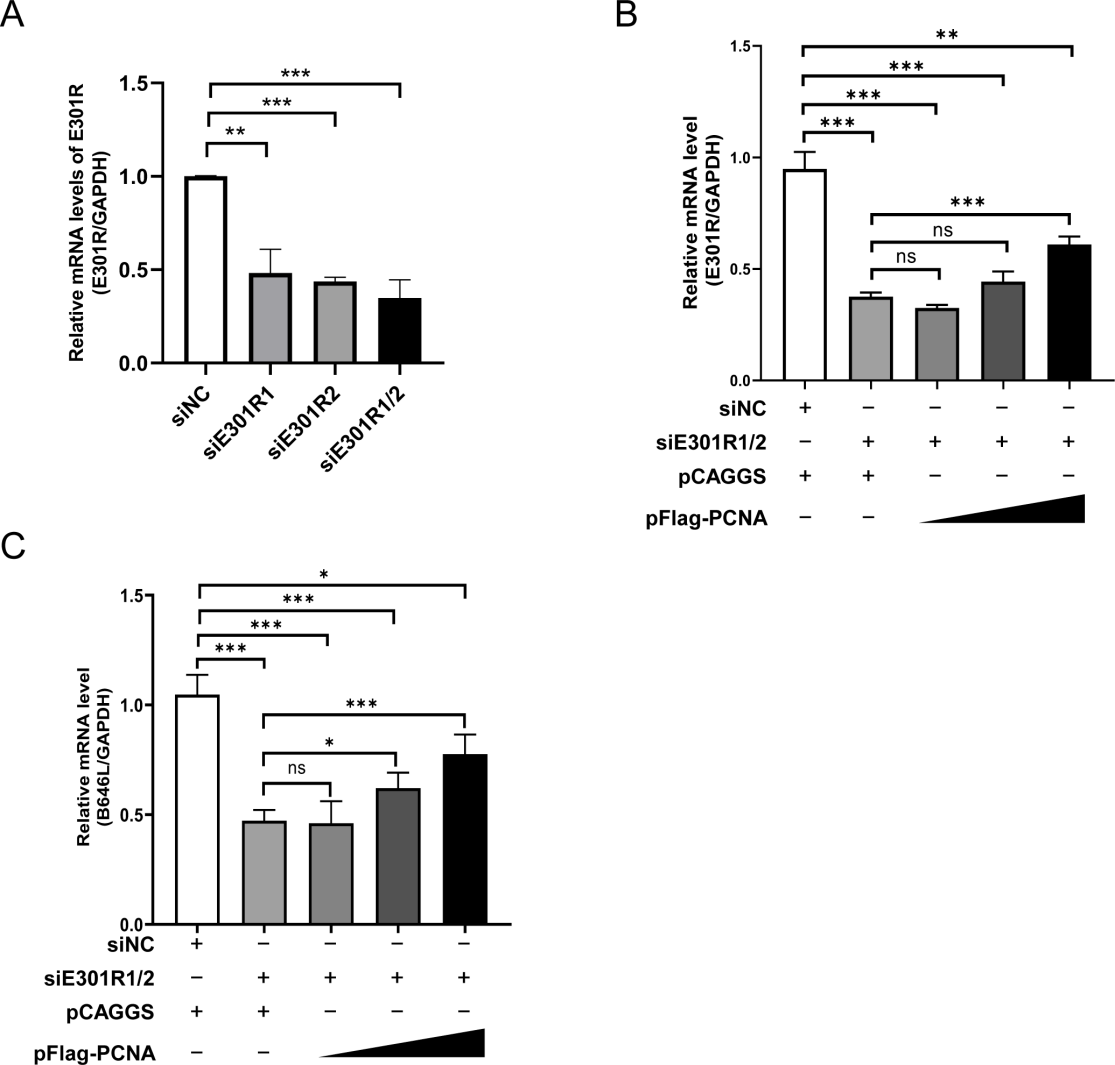
Overexpression of the porcine PCNA partially restores the African swine fever virus (ASFV) infection with *E301R* knockdown. (**A**) Knockdown efficiency of the siRNAs targeting *E301R*. HEK293T cells were transfected with 200 nM single or pooled siRNA(s) targeting *E301R* for 6 hours, followed by infection with ASFV-P121 at a multiplicity of infection (MOI) of 1. At 48 hours postinfection, the knockdown efficiency of siRNA(s) was determined by a relative reverse transcription-quantitative PCR (2^−ΔΔCT^ method). (**B and C**) Relative mRNA levels of the viral genes *E301R* and *B646L*. HEK293T cells in 24-well plates were co-transfected with siE301R1/2 (200 nM) and pFlag-PCNA of different amounts (0, 0.5, 1.0, and 2.0 µg) for 6 hours and then infected with ASFV-P121 at an MOI of 1 for 48 hours. The intracellular relative mRNA levels of the viral genes *E301R* (B) and *B646L* (C) were quantified as described above. The target gene expression was normalized to the expression of the human *GAPDH* gene. *P < 0.05, **P < 0.01, ***P < 0.001; ns, not significant, *P* > 0.05.

Considering that overexpression of pE301R restored the viability of the PCNA-knockdown cells, *E301R* could not be deleted from the ASFV genome by standard genetic manipulation. To further verify the sliding clamp-like function of pE301R, we tested whether PCNA can compensate for the function of the ASFV pE301R. First, we evaluated the knockdown efficacy of siRNA targeting the *E301R* gene and revealed that the pooled siE301R1/2 (equal amounts of each siRNA) displayed the optimal knockdown efficiency (Fig. 7A). Subsequently, HEK293T cells were co-transfected with pFlag-PCNA and siE301R1/2 for 6 hours and then infected with ASFV-P121 at an MOI of 1. At 48 hpi, the cell pellets were collected for the examination of viral transcripts of *E301R* and *B646L*. The results showed that *E301R* knockdown inhibited ASFV replication, partially restored by the ectopically expressed porcine PCNA (Fig. 7B and C). Collectively, the data show that pE301R functions as a sliding clamp.

### The PCNA-specific inhibitor or small interfering RNAs against *E301R* significantly inhibit ASFV replication

**Fig 8:**
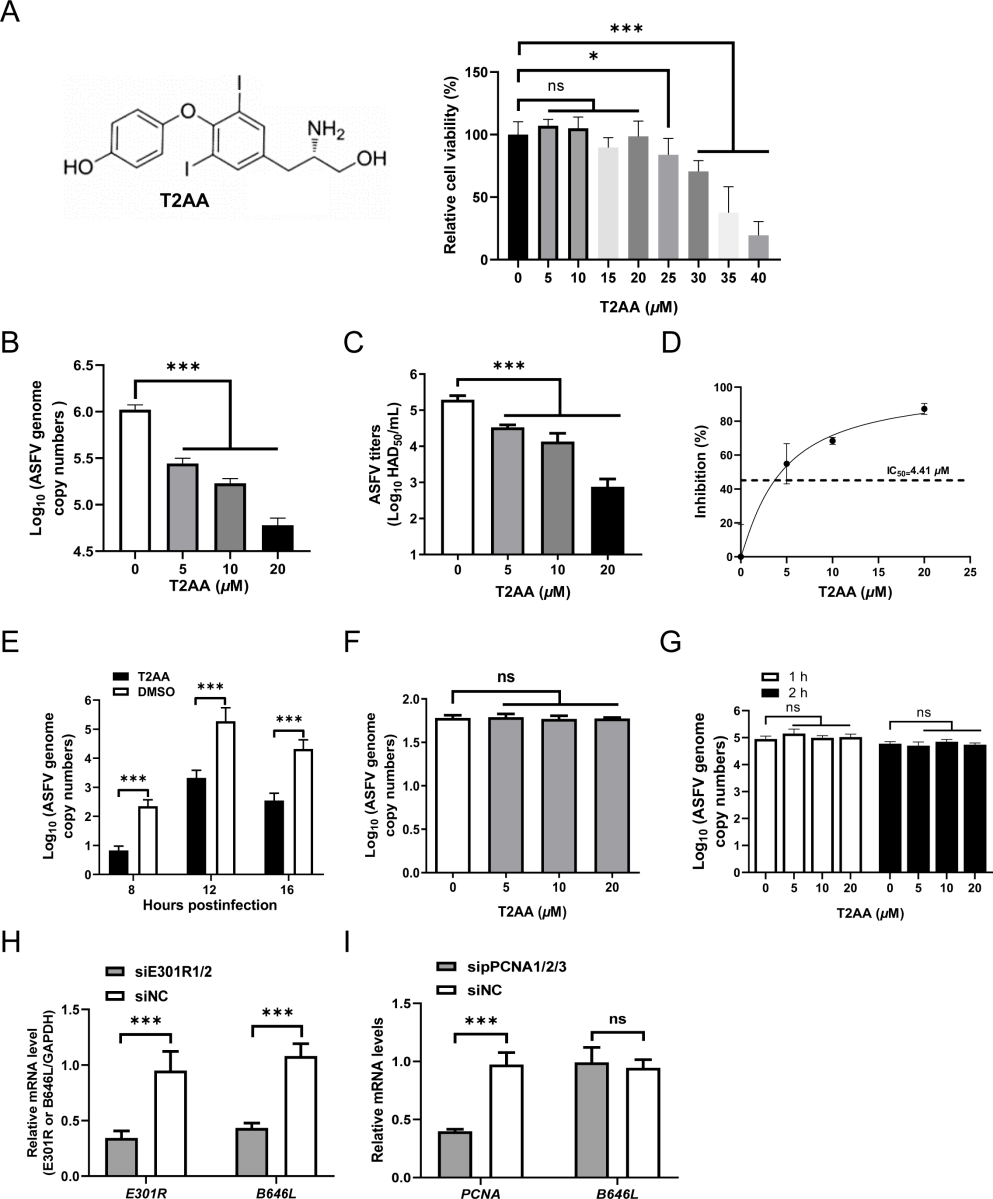
PCNA-specific inhibitor or siRNA against *E301R* significantly inhibits African swine fever virus (ASFV) replication. (**A**) Cell viability assay. Primary porcine alveolar macrophages (PAMs) were treated with the PCNA-specific inhibitor T2 amino alcohol (T2AA) of various concentrations (0, 5, 10, 15, 20, 25, 30, 35, and 40 µM) for 48 hours, and the cell viability was examined by using a CellTiter-Glo kit. (B–D) T2AA inhibited ASFV growth. PAMs were inoculated with ASFV at a multiplicity of infection (MOI) of 0.1 in the presence of T2AA at different final concentrations (0, 5, 10, and 20 µM). At 48 hours postinfection (hpi), the supernatants were collected to examine the viral genome copy numbers (**B**) and the progeny virus titers (**C**), and the IC_50_ of T2AA was determined as 50% inhibition of the viral copy numbers in the supernatants (D). (**E**) Effects of T2AA on ASFV genomic replication. PAMs were infected with ASFV-WT at an MOI of 0.1 for 1 hour at 4°C, and then the cells were shifted to 37°C for 1 hour. After washing with phosphate-buffered saline, the cells were treated with 20 µM T2AA, and the intracellular viral genome copies were analyzed at 8, 12, and 16 hpi using a quantitative PCR (qPCR). (**F**) Effects of T2AA on virus entry. PAMs were infected with ASFV-WT at an MOI of 0.1 at 4°C for 1 hour to allow virus attachment, and then the cells were shifted to 37°C for 1 hour in the presence of the inhibitor. The intracellular ASFV genomic copy numbers were quantified by a qPCR. (**G**) Effects of T2AA on virus release. PAMs were infected with ASFV at an MOI of 0.1 for 24 hours. Then, the supernatants were discarded and replaced with fresh RPMI 1640 containing different concentrations of T2AA. The supernatants were collected after 1 and 2 hours, and the viral genome copies were determined as described above. (**H**) *E301R* knockdown by specific siRNAs impairs ASFV replication. PAMs were transfected with 200 nM siRNAs against *E301R* for 6 hours. The cells were infected with ASFV-WT at an MOI of 0.1. At 48 hpi, the cells were used to quantify the mRNA level of the ASFV *E301R* gene by the relative RT-qPCR (2^−ΔΔCT^ method). The target gene expression was normalized to the expression of the porcine *GAPDH* gene. (**I**) Knockdown of PCNA cannot inhibit ASFV replication. PAMs were transfected with 200 nM pooled siRNAs against the porcine PCNA (sipPCNA1/2/3) for 6 hours. The cells were infected with ASFV-WT, and the mRNA level of the ASFV *B646L* or the *PCNA* gene was determined as described above. *P < 0.05, ***P < 0.001; ns, not significant, *P* > 0.05.

To determine whether pE301R could be an antiviral target, T2 amino alcohol (T2AA), a PCNA-specific inhibitor, was used to verify its effects on the ASFV replication. We first determined the maximum noncytotoxic concentration of T2AA in PAMs, and the results demonstrated that a concentration below 20 µM showed no effects on the cell viability (Fig. 8A). Furthermore, T2AA treatment resulted in the reduction of ASFV genomic copies (Fig. 8B) and viral titers (Fig. 8C) in a dose-dependent manner. Notably, the inhibition rate of viral replication was dose-dependent as calculated using the GraphPad Prism software (version 8.0), with an IC_50_ of 4.41 µM (Fig. 8D). To determine which stage(s) of the ASFV life cycle is inhibited by T2AA, the PAMs infected with ASFV were treated with T2AA at different stages of the virus life cycle. We found that the virus genomic copies were markedly reduced by T2AA treatment at viral genome replication stage (Fig. 8E) but not virus entry (Fig. 8F) or release (Fig. 8G) stage. These results indicated that T2AA exhibits antiviral activities at the genomic replication stage, consistent with the identified function of pE301R.

To exclude the effects of cellular PCNA targeting by T2AA on the ASFV replication, PAMs transfected with siRNAs against porcine *PCNA* or *E301R* were infected with ASFV-WT at an MOI of 0.1 for 48 hours. The results showed that the knockdown of *E301R* significantly suppressed the transcriptional level of *B646L* (Fig. 8H), whereas the knockdown of PCNA did not inhibit ASFV replication (Fig. 8I), indicating that the cellular PCNA does not participate in ASFV replication. Overall, the data demonstrate that T2AA targets pE301R (the viral PCNA analog) but not the cellular PCNA to exhibit the antiviral activities.

## DISCUSSION

Very little is known about the molecular mechanisms underlying the genomic replication in large DNA-enveloped viruses including ASFV and poxviruses. Large DNA viruses mainly encode the enzymes and proteins involved in DNA replication for the virus life cycle. It has been shown that poxviruses encode several enzymes involved in genomic replication, including DNA polymerase, PF, helicase, thymidine kinase (TK), ribonucleotide reductase, and dUTPase. Although the corresponding poxvirus mutants defective in the synthesis of deoxyribonucleotides are viable, they are generally attenuated *in vivo* (27). Similarly, the enzymes and proteins encoded by ASFV are directly involved in the viral genome replication, including a DNA polymerase (pO174L) and a sliding clamp-like protein (pE301R) that clamps the polymerase to slide along the genome. The ASFV mutants lacking several key enzymes, including TK or dUTPase, are viable but with reduced viral replication *in vitro* or *in vivo*. The halovirus фCh1 *ORF59*-encoded PCNA is crucial but not essential for the virus life cycle. The deletion of the фCh1 *ORF59* resulted in a significant reduction in the virus titer, whereas overexpression of *ORF59* revealed that the modification of the GTG start codon to ATG changed the life cycle in terms of production of progeny virus particles (28). However, the *E301R*-deleted ASFV mutant was non-viable, indicating that *E301R* is essential for the ASFV life cycle.

ASFV DNA replication begins in perinuclear viral factories at around 6 hpi through an earlier stage of nuclear DNA synthesis (11), which is consistent with the time points of *E301R* transcription initiation, indicating that pE301R functions in the stage of viral DNA replication. PCNA also interacts with several proteins, which can be grouped into three categories: DNA replication (DNA polymerase δ), DNA repair, and cell cycle regulation. We showed that pE301R interacts with the ASFV DNA polymerase pO174L and mainly locates in the viral factories. In addition, we demonstrated that pE301R interacts with the ASFV genome.

In this study, we revealed that the ASFV pE301R is a viral analog of the sliding clamp. The structure of pE301R reveals a ring-shaped head-to-tail homotetramer that can be classified as a new member of DNA sliding clamps. They have a common topological overall structure composed of two similar globular domains except for the bacterial β clamps with three domains (5, 29). The PCNAs from eukaryotes and archaebacterial form ring-shaped trimmers with head-to-tail contacts as well as Gp45 from the T4 and RB69 bacteriophages (23, 29 – 31), whereas bacterial β clamps form ring-shaped dimers with head-to-tail contacts (32) (Fig. 3D). On the other hand, the herpesvirus polymerase PFs also display different molecular assemblies, such as monomers (HSV-1 UL42) and head-to-head dimers (HCMV UL-44 and KSHV PF-8) (33 – 35). From these comparisons, we conclude that the oligomerization architecture and assembly manner of ASFV pE301R are distinct from known DNA sliding clamps or PFs. To map the key amino acid(s) responsible for the homotetramerization, we also attempted several single or double mutations of the residues on the interface of the tetramer ring. The resulting mutants have a nearly identical elution volume to the wild-type in a size-exclusion column, indicating that these mutations do not disrupt the tetramer formation. We also tried to generate several mutants with multiple mutations, but, unfortunately, the mutants formed aggregates which were unsuitable for further experiments. The tetramer ring likely represents a stable status that is stabilized by multiple H-bonds and hydrophobic contacts as resolved in the crystal structure (Fig. 4B). Interestingly, we demonstrated that two major types of oligomers (tetramers and dimers) were detected by native PAGE in the ASFV-infected PAMs and AUC analysis *in vitro*. The two oligomers may represent the ring-shaped homotetramers similar to eukaryotic PCNAs and the homodimers like the PFs of HCMV and KSHV, respectively. The two types of pE301R oligomers might function in different stages of the ASFV life cycle, while the precise mechanisms need to be investigated in the future. During the preparation of our article, the crystal structure of pE301R in full length was reported (36). Interestingly, the pE301R structure adopts the *R*3 space group which formed a ring-shaped head-to-tail homotrimer in the crystal lattice, whereas the *I*4 space group of our structure generates the ring-shaped head-to-tail homotetramer. It should be noted that, although the negative staining of pE301R in the recent work showed a ring-shaped architecture, it is difficult to observe the exact number of subunits within this ring at such a low resolution. Our AUC data support the tetramer (rather than trimer) formation in solution, a convincing method to determine the exact molecular weight or oligomeric state of certain proteins. In addition, both their negative staining and our AUC analysis revealed higher-order assemblies *in vitro*, indicating variable oligomers of pE301R may be needed to perform its multiple functions in ASFV.

As the DNA sliding clamps tether their cognate DNA polymerases to the DNA template to increase their processivity, they play important roles in DNA replication and repair, regulation of cell cycle and apoptosis, and cell proliferation indicator (37). We revealed that *PCNA* knockdown resulted in the reduction of cell viability, whereas the ectopic expression of pE301R compensated the cell viability induced by PCNA downregulation, suggesting that pE301R exhibits a sliding clamp-like function in mammalian cells. Since the *E301R* gene-deleted ASFV is non-viable, we analyzed in detail the ASFV replication by knocking down *E301R* and demonstrated that the virus replication was reduced, revealing novel insights into the importance of pE301R for the virus life cycle. Importantly, the inhibitor T2AA targeting the viral PCNA analog exhibits a robust antiviral effect. Therefore, pE301R could be used as a potential target for the antiviral strategies. PCNA has been shown to complement some of the modulatory functions of pE301R during viral infection. We also tried to generate an ASFV mutant with *E301R* substituted with *PCNA*, but the mutant virus could not be rescued, and the downregulated ASFV infection upon *E301R* knockdown was partially restored by the ectopically expressed PCNA, indicating that the PCNAs from other species could partially compensate the function of pE301R. We presumed that the structural difference between PCNA and pE301R could reduce the binding affinity of DNA polymerase or DNA with PCNA. It has been shown that EBV utilizes the cellular PCNA and the viral polymerase PF BMRF1 for the latent and lytic viral replication, respectively; however, the EBV genome is amplified 100- to 1000-fold by the viral replication machinery compared with the cellular replication machinery (38). Viruses likely utilize viral PCNA analogs that are more effective in facilitating viral replication than do the cellular PCNAs. Interestingly, we demonstrated that PCNA partially participated in ASFV infection in the absence of *E301R* (Fig. 7C). In contrast, knockdown of the cellular *PCNA* did not impair ASFV infection in PAMs (Fig. 8I). We presumed that ASFV replication preferentially depends on its own sliding clamp-like protein but not cellular PCNA. Once the viral sliding clamp-like protein is knocked down, the cellular PCNA can be hijacked by ASFV to compensate for its functions partially. Thus, the knockdown of cellular PCNA cannot impair ASFV replication.

Structural comparisons of pE301R with the human and *P. furiosus* PCNAs revealed that the major differences are the various conformations of the IDCL loops that function in recruiting partner proteins (Fig. 3B). A putative nuclear localization signal (NLS) in the long N-terminal loop (residues 6–19) of pE301R is absent in the eukaryotic and archaebacterial PCNAs, while a putative variable nuclear export signal (NES) is located at the C-terminus (residues 278–301) (Fig. 3C), which is different from the PCNAs of different species. The role of the N-terminal extension of pE301R (residues 1–35, including the NLS) was recently supposed to prevent higher-order assemblies that may be not suitable for DNA-binding and to maintain the ring-shape trimer (although such oligomer was not observed in our study) (36). We demonstrated that pE301R was localized in the cytoplasm and, occasionally, in the nucleus using laser confocal microscopy. A recent study also revealed that pE301R is distributed in the cytoplasm and nucleus of the pE301R plasmid-transfected cells using western blotting analysis (39). We presumed that the nuclear translocation of pE301R occurs at the early stage of ASFV infection. However, we failed to detect pE301R at the early stage due to the low-level expression using a homemade antibody (data not shown). The pE301R nuclear localization should be confirmed using a high-affinity antibody in the future. ASFV DNA replication has also been detected in the nucleus in the early stage of the virus life cycle, and the virus genome is translocated to the viral factories in the cytoplasm for replication (11). The NLS and NES of pE301R are likely to regulate the entry or exit of the virus genome into or off the nucleus. The *E301R NLS*-deleted mutant ASFV needs to be generated to define the involvement of the NLS in viral replication.

In summary, we demonstrated for the first time that pE301R displays the sliding clamp-like function in the ASFV genomic replication. Our findings not only unveil the function of pE301R in the ASFV life cycle but also provide a novel target for developing antiviral strategies to combat ASF.

## MATERIALS AND METHODS

### Cells, viruses, and antibodies

HEK293T cells were grown in Dulbecco’s modified Eagle’s medium (catalog no. C11995500BT, Gibco) supplemented with 10% fetal bovine serum (FBS) (catalog no. 12,007C, Sigma-Aldrich). PAMs were cultured with RPMI 1640 medium (catalog no. C11875500BT, Gibco) supplemented with 10% FBS and 2% antibiotics-antimycotics (100 IU/mL penicillin, 100 mg/mL streptomycin, and 25 mg/mL amphotericin B) (catalog no. 15240-062, Gibco).

The Pig/HLJ/2018 strain (ASFV-WT) was isolated from field pig samples in China, as described previously (40). ASFV-P121, a cell-adapted ASFV strain, replicates efficiently in HEK293T cells (41).

### Construction of plasmids

The *E301R* gene with a Flag tag at its 3′ end was amplified from the genome of ASFV (GenBank accession number: MK333180.1) by PCR and subsequently cloned into the pCAGGS vector (Addgene), resulting in the expression plasmid pFlag-E301R. Likewise, the HA- and Myc-tagged E301R fragments were subcloned into the pCAGGS vector (Addgene), creating pHA-E301R and pMyc-E301R, respectively. The Flag-tagged *O174L* gene was cloned into pCAGGS, resulting in pFlag-O174L. The *E301R* gene with a strep tag at its 3′ end was amplified by PCR from the ASFV genome and then inserted into the pCold-I vector to generate pCold-E301R. All the primers used for gene amplification in the study are listed in Table 2.

**TABLE 2:** Primers used in this study

Primers	Sequence (5′ → 3′)	Description
pCAGGS-E301R-Flag-F	CATCATTTTGGCAAAGAATTCGCCACCATGGATTACAAGGATGACGACGATAAGTCTGAAGATATTCGTCGTGGTC	For the gene *E301R*
pCAGGS-E301R-Flag-R	TTGGCAGAGGGAAAAAGATCTCTATATCGTGGTGTTCAAGGTAATCG	
pCAGGS-E301R-HA-F	CATCATTTTGGCAAAGAATTCGCCACCATGTACCCATACGACGTCCCAGACTACGCTTCTGAAGATATTCGTCGTGGTC	For the gene *E301R*
pCAGGS-E301R-HA-R	TTGGCAGAGGGAAAAAGATCTCTATATCGTGGTGTTCAAGGTAATCG	
pCAGGS-E301R-Myc-F	CATCATTTTGGCAAAGAATTCGCCACCATGGAGCAGAAACTCATCTCTGAAGAGGATCTGTCTGAAGATATTCGTCGTGGTC	For the gene *E301R*
pCAGGS-E301R-Myc-R	TTAGCAGAGGGAAAAAGATCTCTATATCGTGGTGTTCAAGGTAATCG	
pCold-I-E301R-F	CGGGATCCATGTCTGAAGATATTCGTCGTGGTC	For expression of the recombinant proteins
pCold-I-E301R-R	CGGAATTCCTACTTCTCGAACTGGGGGTGGGACCATATCGTGGTGTTCAAGGT	
pCAGGS-O174L-Flag-F	CATCATTTTGGCAAAGAATTCGCCACCATGTTAACGCTTATTCAAGGAAAAAA	For the gene *O174L*
pCAGGS-O174L-Flag-R	ATCGTCTTTGTAGTCCTCGAGTAAACGTTTCTTAGGTATGCGATACG	
pCAGGS-NP419L-Flag-F	ATCATTTTGGCAAAGAATTCGCCACCATGCTAAATCAATTTCCTGGGC	For the gene *NP419L*
pCAGGS-NP419L-Flag-R	ATCGTCTTTGTAGTCCTCGAGAATGATTTCTAAAACACTTATCGGTTCA	
Q-GAPDH-pF	GAAGGTCGGAGTGAACGGATTT	RT-qPCR for porcine *GAPDH*
Q-GAPDH-pR	TGGGTGGAATCATACTGGAACA	
Q-GAPDH-hF	GACACCCACTCCTCCACCTTT	RT-qPCR for human *GAPDH*
Q-GAPDH-hR	ACCACCCTGTTGCTGTAGCC	
Q-E301R-F	GTCGCAGGTGTTCCAGATAAA	RT-qPCR for *E301R*
Q-E301R-R	GGCATCCATTTCCGATTGAAAG	
Q-hPCNA-F	GGATACCTTGGCGCTAGTATTT	RT-qPCR for human *PCNA*
Q-hPCNA-R	CACAGCTGTACTCCTGTTCTG	
Q-pPCNA-F	GGAGGAAGCAGTTACCATA	RT-qPCR for pig *PCNA*
Q-pPCNA-R	TGTGACTGTAGGAGAGAGT	
Q-B646L(p72)-F	CTGCTCATGGTATCAATCTTATCGA	RT-qPCR, qPCR and ChIP-qPCR for *B646L*
Q-B646L(p72)-R	GATACCACAAGATC(AG)GCCGT	
Q-CP204L(p30)-F	AGCGGTCGTAACAATTCTACC	RT-qPCR for *CP204L*
Q-CP204L(p30)-R	AGTTGTGTTTCATGCGGGTAG	
ChIP-Q-MGF360-1L-F	GGGCTGACATTAATCGGGCA	ChIP-qPCR for *MGF360-1L*
ChIP-Q-MGF360-1L-R	GGCTCGCTATTTCCATGCTC	
ChIP-Q-F778R-F	GCACTATCACGAACTCCAATCT	ChIP-qPCR for *F778R*
ChIP-Q-F778R-R	CACGGTAGTCGTAGCCATTT	
ChIP-Q-O174L-F	AATGACTTACGCTCCCGACTT	ChIP-qPCR for *O174L*
ChIP-Q-O174L-R	TTTCGTTCTCCGCAGACTTTT	
ChIP-Q-NP419L-F	AAAAGACGCCGTTGCTGAAAT	ChIP-qPCR for *NP419L*
ChIP-Q-NP419L-R	TTGAATGCCTGATGGACTACC	
ChIP-Q-DP60R-F	GGCGGCGTAACACCAGTTAT	ChIP-qPCR for *DP60R*
ChIP-Q-DP60R-R	GCCGCGGCCGGAAATA	

### Protein expression and purification

The *E301R* gene was cloned into pET-28at-plus (introducing an N-terminal tobacco etch virus protease cleavage site constructed by our laboratory). The recombinant pE301R was expressed in *Escherichia coli* BL21(DE3) and purified as described recently (42), with some modifications for lysine-methylation of pE301R according to a previously established method (43). The recombinant pE301R was further purified by gel filtration (Superdex 200; GE Healthcare, USA) equilibrated in a buffer containing 20 mM HEPES (pH 7.2), 300 mM NaCl, and 2 mM dithiothreitol using an ÄKTA Purifier System (GE Healthcare). Se-Met-substituted pE301R was produced and purified as described previously (44).

### Protein crystallization

The initial crystallization condition for pE301R was determined under the 19# crystallization condition of the SaltRx kit (catalog no. HR2-136, Hampton Research) with the sitting drop vapor diffusion method at room temperature for 3 days. The crystal quality was improved by optimizing the precipitant concentration and buffer. The optimal crystal was obtained in solution 0.1 M Bis-Tris (pH 7.0) and 1 M sodium citrate after 3–4 days.

### Data collection, crystal structure determination, and refinement

The diffraction data from a single crystal of the Se-Met-substituted protein were collected on the beamline station BL18U1 of the Shanghai Synchrotron Radiation Facility (SSRF) using an EIGER pixel detector at a wavelength of 0.9788 Å. The total oscillation was 360° with 1° per image, and the exposure time was 0.3 s. Before data collection, the crystals were soaked in the reservoir solution supplemented with 20% (vol/vol) glycerol for a few seconds and then flash-frozen in liquid nitrogen. All the data were processed by XDS (45). The Se-Met crystal structure of pE301R was determined by the single-wavelength anomalous dispersion method. The selenium atoms were located by the program Shelxd and then used to calculate the initial phases in Shelxe (46). The phases from Shelxe were improved in Resolve (47) and then used in Buccaneer (48) for model building.

The diffraction data from a single crystal from native protein samples were collected on the beamline station BL17U1 of SSRF using a Pilatus 6M detector at a wavelength of 0.9788 Å. The total oscillation was 360° with 1° per image, and the exposure time was 1 s per image. Before data collection, the crystals were soaked in the reservoir solution supplemented with 20% (vol/vol) glycerol for a few seconds and then flash-frozen in liquid nitrogen. All the data were processed by XDS as described previously (45). The initial phases were calculated using the program PHASER, with the initial phase above as the search model. The structure was refined with the program Phenix.refine (49) and manually corrected in Coot (50). The qualities of the final models were validated with the program MolProbity (51). Refinement statistics and model parameters are shown in Table 1. The program PyMOL (https://pymol.org/2/) was used to generate the structural images.

### Co-IP assay

HEK293T cells in 12-well plates were co-transfected with 3 µg each of pFlag-E301R and pMyc-O174L. The cells were lysed with NP-40 lysis buffer (catalog no. P0013F, Beyotime) containing 1 mM phenylmethylsulfonyl fluoride (catalog no. ST506-2, Beyotime) at 48 hours posttransfection (hpt), followed by centrifugation at 12,000 × *g* for 20 minutes at 4°C to remove the cell debris. The lysates were precleared with protein G-agarose (catalog no. 11243233001, Merck) and incubated with anti-Flag M2 magnetic beads (catalog no. M8823, Sigma-Aldrich) for 4 hours at 4°C. After washing with cold phosphate-buffered saline (PBS), the bounded proteins were analyzed by SDS-PAGE and western blotting using anti-Myc (catalog no. C3956, Sigma-Aldrich) or anti-Flag (catalog no. F7425, Sigma-Aldrich) PAbs.

### Laser confocal microscopy

PAMs were infected with ASFV at an MOI of 1 for 24 hours. The cells were fixed with 4% paraformaldehyde and permeabilized with 0.15% Triton X-100 for 20 minutes. Subsequently, the cells were incubated with the mouse anti-pE301R (1:500) or swine anti-p54 PAbs (1:500) at 37°C for 1 hour. After washing three times with PBS, the cells were incubated with an Alexa Fluor 633 goat anti-mouse IgG (catalog no. A-21050, Thermo Scientific) anda rabbit anti-pig IgG (whole molecule)-fluorescein isothiocyanate (catalog no. F1638, Merck) for 1 hour. The cells were incubated with 4,6-diamidino-2-phenylindole for 15 minutes and examined using a Zeiss confocal system.

### Cell viability assay

The cell viability of the HEK293T cells treated with the ectopically expressed pE301R upon PCNA knockdown was analyzed using a CellTiter-Glo kit (catalog no. G7572, Promega) according to the manufacturer’s instructions.

To determine the maximum non-cytotoxic concentration of T2AA for PAMs, T2AA of different concentrations was added to PAMs for 48 hours. Subsequently, the cell viability was tested using the CellTiter-Glo kit.

### RNA interference assay

The siE301Rs and the scramble control siRNA were synthesized by GenePharma. PAMs grown in 24-well plates were transfected with 200 nM pooled siE301Rs using the X-tremeGENE siRNA transfection reagent (catalog no. 4476115001-1, Roche). At 6 hpt, the cells were infected with ASFV-WT at an MOI of 0.1. At 48 hpi, the cells were harvested to examine viral genome copies, and the mRNA transcription of the viral *B646L* or *E301R*.

The sihPCNAs were synthesized by GenePharma. HEK293T cells grown in 96-well plates were transfected with 200 nM pooled siRNAs using the X-tremeGENE siRNA transfection reagent. Cell viability was analyzed at 24 hpt. The expression of PCNA was determined by western blotting using an anti-PCNA MAb (catalog no. ab29, Abcam). The sequences of the siRNAs are shown in Table 3.

**TABLE 3:** The sequences of the siRNAs used in this study

Names	Sequence (5′ → 3′)	Description
siE301R1-F	GGAGUGCACGUAUCAGAUUTT	For the gene *E301R*
siE301R1-R	AAUCUGAUACGUGCACUCCTT	
siE301R2-F	GCAGGUGUUCCAGAUAAAUTT	
siE301R2-R	AUUUAUCUGGAACACCUGCTT	
sipPCNA1-F	GGUGCUGGAAGCGCUUAAATT	For the gene *PCNA* of pig
sipPCNA1-R	UUUAAGCGCUUCCAGCACCTT	
sipPCNA2-F	GGUGAAUUUGCACGUAUAUTT	
sipPCNA2-R	AUAUACGUGCAAAUUCACCTT	
sipPCNA3-F	GGAGAACUCGGAAAUGGAATT	
sipPCNA3-R	UUCCAUUUCCGAGUUCUCCTT	
sihPCNA1-F	GCUCCAUCCUCAAGAAGGUTT	For the gene *PCNA* of human
sihPCNA1-R	ACCUUCUUGAGGAUGGAGCTT	
sihPCNA2-F	GCGUGAACCUCACCAGUAUTT	
sihPCNA2-R	AUACUGGUGAGGUUCACGCTT	
sihPCNA3-F	GCACCAAACCAGGAGAAAGTT	
sihPCNA3-R	CUUUCUCCUGGUUUGGUGCTT	
siNC-F	UUCUCCGAACGUGUCACGUTT	Scramble control siRNA
siNC-R	ACGUGACACGUUCGGAGAATT	

### Quantitative PCR (qPCR)

The viral genome copies of ASFV-infected cells collected at various time points were determined by a qPCR (52). According to the manufacturer’s instructions, ASFV genomic DNA was extracted from the cell supernatants using the MagaBio plus virus DNA purification kit (catalog no. 9109, BioFlux). The viral genome copies were quantified by a qPCR as described previously (53).

The mRNA level of viral *E301R* or *B646L* was quantified by RT-qPCR. The total RNA was extracted from the ASFV-infected cells with Simply P Total RNA extraction kit (catalog no. BSC52M1, BioFlux) and treated with DNase I to remove potential genomic DNA contaminants. The isolated RNA was then reverse transcribed to cDNA with FastKing gDNA Dispelling RT SuperMix (catalog no. KR118-02, Tiangen) according to the manufacturer’s protocols. The mRNA transcriptional level was quantified by relative RT-qPCR with SYBR Premix Ex Taq II (catalog no. RR390B, TaKaRa). Relative fold changes in mRNA transcription were determined by the threshold cycle (2^−ΔΔCt^) method (54). The primers for amplifying the *E301R*, *B646L*, and *GAPDH* genes are listed in Table 2.

### Inhibitor treatment assay

To examine the effects of the PCNA-specific inhibitor T2AA on ASFV replication, PAMs were infected with ASFV-WT at an MOI of 0.1. After washing three times with PBS, the cells were further cultured for 48 hours in the presence of T2AA (catalog no. CAC-21921-5, Cayman) or dimethyl sulfoxide (catalog no. D2650, Sigma-Aldrich). The supernatants or pellets were collected to determine the viral genome copy numbers or mRNA transcripts of the viral genes.

### Virus titration assay

PAMs grown in 96-well plates were infected with serially 10-fold-diluted ASFV for 96 hours at 37°C. Around 10^6^ swine erythrocytes in PBS were added to the cells, and hemadsorption was examined under a microscope at 120 hpi.

### Chromatin immunoprecipitation-quantitative PCR

ChIP assay was conducted using the SimpleChIP enzymatic chromatin IP kit (catalog no. 9003S, Cell Signaling Technology) according to the manufacturer’s instructions with sight modification. Briefly, 4 × 10^7^ cells in 10-cm culture dishes were incubated with 1% formaldehyde for 10 minutes at room temperature to cross-link proteins to DNA. The cross-linking was then quenched with 0.125 M glycine for 5 minutes. Subsequently, the cells were centrifuged at 2000 × *g* for 5 minutes at 4°C, lysed with SDS lysis buffer containing a protease inhibitor cocktail, and sonicated to shear the DNA. The sonicated DNA-protein complexes were incubated with anti-Flag PAbs (catalog no. F7425, Sigma-Aldrich) or control IgG (catalog no. 9003S#2729, Cell Signaling Technology) antibodies for 4 hours at 4°C and then incubated with the protein G magnetic beads (catalog no. 9003S#9006, Cell Signaling Technology) for 2 hours at 4°C. The beads were washed three times with wash buffer A (low-salt wash) and once with wash buffer B (high-salt wash). Next, the beads were eluted with 150 µL of elution buffer followed by incubation at 65°C for 10 hours to reverse cross-linking. Afterwards, the DNA was purified with a spin column and analyzed by qPCR using ChemQ SYBR qPCR master mix (catalog no. Q311-02, Vazyme).

### Analytical ultracentrifugation

The sedimentation velocity was measured using a Beckman Optima XL-I analytical ultracentrifugation (Beckman-Coulter Instruments) with a Ti rotor at 20°C. The pE301R protein concentration was adjusted to absorption of around 0.60 at 280 nm. The SEDFIT program was used to analyze the sedimentation coefficient (55).

### Statistical analysis

All statistical analyses were determined using the SPSS software (version 23.0; SPSS Software, Inc.). Student’s *t-*test was used to assess statistical significance (*P < 0.05, **P < 0.01, ***P < 0.001; ns, not significant, *P* > 0.05). Error bars denote the standard deviations of the means.

## Data Availability

The atomic coordinates and structure factors of pE301R have been deposited with the RCSB PDB with the identifier code 8ITE.
